# Correlation study between flash dual source CT perfusion imaging and regional lymph node metastasis of non-small cell lung cancer

**DOI:** 10.1186/s12885-020-07032-8

**Published:** 2020-06-12

**Authors:** Tingting Huang, Hui Sun, Xianli Luo, Xuemei Zhang, Kaiyuan Jin, Feng Wang, Lv Sun, Nianlan Cheng, Shuo Wu, Qin Lou, Bangguo Li

**Affiliations:** 1 Department of Radiology, Affiliated Hospital of Zunyi Medical University, No.149, Dalian Road, Zunyi City, Guizhou Province China; 2 Department of Radiology, The Third Affiliated Hospital of Qiqihar Medical University, Qiqihar, Heilongjiang Province China; 3 Department of Radiology, Affiliated Hospital of Guangdong Medical University, Zhanjiang, Guangdong Province China; 4 Department of Radiology, Guizhou Provincial People’s Hospital, Guiyang, Guizhou Province China

**Keywords:** Non-small lung cancer, Lymph node metastasis, Computed tomography perfusion imaging, Microvessel density, Luminal vessels

## Abstract

**Background:**

To explore the correlation of flash dual source computed tomography perfusion imaging (CTPI) and regional lymph node metastasis of non-small cell lung cancer (NSCLC), and to evaluate the value of CT perfusion parameters in predicting regional lymph node metastasis of NSCLC.

**Methods:**

120 consecutive patients with NSCLC confirmed by postoperative histopathology were underwent flash dual source CT perfusion imaging in pre-operation. The CT perfusion parameters of NSCLC, such as blood flow (BF), blood volume (BV), mean transit time (MTT) and permeability (PMB) were obtained by the image post-processing. Then microvessel density (MVD), luminal vascular number (LVN), luminal vascular area (LVA) and luminal vascular perimeter (LVP) of NSCLC were counted by immunohistochemistry. These cases were divided into group A (patients with lymph node metastasis, 58 cases) and group B (patients without lymph node metastasis, 62 cases) according to their pathological results. The CT perfusion parameters and the microvessel parameters were contrastively analysed between the two groups. Receiver operating characteristic (ROC) curve was used to assess the diagnostic efficiency of CT perfusion parameters in predicting regional lymph node metastasis of NSCLC in pre-operation.

**Results:**

Group A presented significantly lower LVA, BF and higher MTT, PMB than Group B (*P* < 0.05), while BV, LVN, LVP and MVD were no significant difference (*P* > 0.05). Correlation analysis showed that BF was correlated with LVA and LVP (*P* < 0.05), while BV, MTT and PMB were not correlated with LVN, LVA and LVP (*P* > 0.05). All the perfusion parameters were not correlated with MVD. According to the ROC curve analysis, when BF < 85.16 ml/100 ml/min as a cutoff point to predict regional lymph node metastasis of NSCLC, the sensitivity, specificity, accuracy, positive predictive value and negative predictive value were 60.8, 81.7, 71.5, 75.6 and 69.5% respectively.

**Conclusion:**

Flash dual source CT perfusion imaging can non-invasively indicate the luminal vascular structure of tumor and BF can be used as one of the important indexes in predicting regional lymph node metastasis of NSCLC in pre-operation.

## Background

Lung cancer has been the leading cause of cancer death, in which non-small-cell lung accounts for more than 80% of the total [1]. So accurate staging of non-small cell lung cancer (NSCLC) is vital for the prognosis of patients. And in this process, lymph node metastasis is one of the important factors [2]. At present, CT diagnosis of NSCLC with or without regional lymph node metastasis is mainly based on the threshold of 10 mm short diameter of lymph node, but there are a certain false negative rate and false positive rate [3, 4]. FDG-PET/CT is currently the most important noninvasive method for diagnosing lymph node metastasis [5–7]. But due to the fact that PET/CT equipment and inspection fees are very expensive, it has not been widely used, especially in China. Tumor angiogenesis plays a key role in the biological behavior of the tumor. It was reported that lymph node metastasis of cervical cancer was closely related to tumor angiogenesis [8]. We know that the tumor angiogenesis indicators including microvessel density (MVD), luminal vessels number (LVN), the luminal vessels area (LVA) and the luminal vessels perimeter (LVP). But these indicators can only be obtained with a invasive examination, and can not monitor changes of the tumor angiogenesis in real time. Computed tomography perfusion imaging (CTPI) can noninvasively reflect tumor angiogenesis, so it is of great value in the study of tumor growth, invasion and metastasis [9]. Previous studies have shown that the correlation between CT perfusion parameters and luminal vascular parameters is better than MVD [9–11]. Flash dual source CT with advanced radiation reduction technology can greatly protect the health and safety of patients, so it is more suitable for CTPI research. Therefore, this study intends to introduce the LVN, LVA and LVP to analyze the microvascular structure of NSCLC and to make correlation analysis with CT perfusion parameters in order to provide the theoretical basis in studying the CT perfusion imaging characteristics of NSCLC with regional lymph node metastasis, then to evaluate better the value of flash dual source CT perfusion parameters in predicting regional lymph node metastasis of NSCLC in pre-operation.

## Methods

### Patients

This study has been approved by the ethics committee of Zunyi Medical University. And each patient or the patient’s family was fully informed and signed the informed consent before performing the pre-operative CT examination. From January 2015 to May 2019, 120 consecutive patients with NSCLC confirmed by histopathology were enrolled in the study according to the following criteria: (i) These cases successfully underwent CT perfusion imaging before surgical resection; (ii) No treatment before CT perfusion imaging, such as chemotherapy or radiotherapy; (iii) The time interval between CT perfusion imaging and surgery was less than 2 weeks; (iv) No serious cardiac, pulmonary or renal dysfunction [9]. Of these cases, 77 patients is males and 43 patients is females. The mean age was 54.35 years (range, 25–74 years). The cases were divided into group A (patient with lymph node metastasis) and group B (patient without lymph node metastasis) according to their pathological results.

### Equipment and methods

CT perfusion scan was performed by using SOMATOM Definition Flash dual source CT scanner (Siemens, Germany) with dose reduction techniques including. CT scan involved two steps. First, an unenhanced CT scan was performed with following scan parameters: 100 kV, 100 mAs, 0.5 pitch, 512 × 512 mm matrixs and slice thickness was 8 mm. CT scanning covered the entire lung. Second, a perfusion CT scan which based on the plain scan was performed after injecting contrast medium (iohexol) 2–4 s in B20f smooth Eva image reconstruction method with following parameters: 80 kV, 120 mAs, 3 mm reconstruction slice thickness (reconstruction interval 2 mm). The scanning range included the upper and lower 3.5 cm of lung tumor and the patient was breathless during the scan. 50 ml iohexol (300 mgI/ml) was injected via an antecubital fossa vein at a flow rate of 6 ml/second, followed by 20 ml of saline flush [9]. In our study, CTDIvol was (60.89 ± 8.27) mGy and DLP is (536.28 ± 45.06) mGy.cm.

### Image post-processing

First, transmitting perfusion imaging data to workstation (Siemens Syngo Multimodality workplace). Then, we use the volume perfusion computed tomography (VPCT) body software to obtain blood flow (BF), blood volume (BV), mean transit time (MTT), permeability (PMB) of NSCLC. We usually choose the thoracic aorta as the reference vessel and set the region of interest (ROI). If there was no thoracic aorta at the lesion level, the carotid artery or brachiocephalic trunk will be selected as the reference vessel. And the maximum level without artifact interference was selected to delineate the ROI around the boundary (2–3 mm from the edge) of the tumor by hand, avoiding the necrosis area, calcification areas and blood vessels. Of 120 cases, 3 cases of necrosis and 1 case of calcification. We asked two similar level radiologists with more than 6 years of experience in interpreting CT perfusion imaging to do the experiments. Neither radiologists knew anything about the patient. We take the average of them as the final data. If the difference between their data is more than 10%, the perfusion data will be recalculated.

### Histopathologic study

After surgery, tumor tissue specimens were fixed in the inflated state by 10% buffered formalin, and embedded in paraffin. The tissue samples (3 μm paraffin slice) that came from the maximum section of tumors which diagnosed to be NSCLC by hematoxylin and eosin staining were immunohistochemically stained by using the PV 2-Step method (PV-9000 2-step plus Poly-HRP Anti-Mouse/Ribbit IgG Detection System, Golden Bridge Co., Beijing, China) with CD34 (Golden Bridge Co., Beijing, China) monoclonal antibody according to the following steps: (i) removing paraffin of the tissue slices; (ii) the slices were incubated in 3% nonionic hydrogen peroxide for 10 min and then washed with phosphate-buffered saline (PBS) three times (2 min each time); (iii) addition of anti-human CD34 monoclonal antibody in a 1:50 dilution for 1 h at room temperature and then washed with PBS three times (2 min each time); (iv) addition of reagent 1(Polymer Helper) for 20 min at room temperature, then washed with PBS three times (2 min each time). Then addition of reagent 2 (Polyperoxidase- anti-mouse/ribbit IgG) as the method of reagent 1; (v) the slices were then stained with DAB solution and subsequently counterstained with hematoxylin; (vi) the slices were then rinsed with distilled water, dried and mounted. The tissue slices were stained with smooth muscle actin (SMA) in the same method as CD34 staining. The control group was set up with PBS solution instead of the first antibody respectively.

### Quantification of histologic microvessel parameters

Each slice was scanned at low magnification (× 40 or × 100) to determine six “hot spot” areas where the number of microvessels was at maximum including four peripheral areas and two center areas. In each area, one field of 0.20 mm^2^ (× 200 magnification, 0.512 mm × 0.383 mm) was chosen randomly for the purposes of counting and measure MVD, LVN, LVP and LVA. MVD were counted in the chosen field at high magnification (× 200). Endothelial cell, cell cluster that was apparently separate from peripheral tissues and branch construct with discrete breaks which highlighted in CD34 staining wrere counted as microvessels. The final MVD value was obtained from the average MVD value in the above six fields. To be considered vascular, it is necessary to have a distinguishable lumen and one or more layers of pericytes and smooth muscle cells stained with smooth muscle actin. Software (image pro plus 6.0) was used for vascular measurements. If the slices exist following situations: (i) the pathological tissue slip off; (ii) there are air bubbles; (iii) uneven staining; (iv) the background is not clean because the non-specific staining, will be excluded. We asked two similar level pathologists with more than 15 years of experience to count each slice respectivel. If the difference between their data is more than 10%, the data will be recalculated. Then we take the average of the two or four results is the final histologic data.

### Statistical analysis

We using statistical software (SPSS 18.0) to perform all statistical analyses. When *P* < 0.05, the difference is considered to indicate a statistical significance. All data, including the perfusion parameters (BF, BV, MTT and PMB) and the microvessel parameters (MVD, LVN, LVP and LVA) were underwent consistency test within inter-observer and intra-observer. All continuous variables were underwent normality test. Normal distribution data were expressed as mean ± SD, and abnormal distribution data were represented as median ± interquartile range (IQR). The two-tailed Student t test or Wilcoxon test was used to compare the perfusion parameters and the microvessel parameters between the group A and the group B. Pearson or Spearman correlation analysis was used to investigate the relationships between the perfusion parameters and the microvessel parameters. The diagnostic efficiency of CT perfusion parameters in predicting regional lymph node metastasis of NSCLC was assessed by using receiver operating characteristic (ROC) curve .

## Results

### General characteristics of patients

Of these cases, group A includes 58 cases (33 cases of adenocarcinoma, 24 cases of squamous cell carcinoma, and 1 case of large cell undifferentiated carcinoma) and group B includes 62 cases (33 cases of adenocarcinoma, 26 cases of squamous cell carcinoma, 2 case of large cell neuroendocrine carcinoma and 1 case of sarcomatoid carcinoma). For pN-stage, there were 62, 23, 35 and 0 cases in pN0, 1, 2 and 3, respectively. For pT-stage, there were 51, 46, 23 and 0 cases in pT1, 2, 3 and 4, respectively. In group A, the primary tumor is from 1 cm to 3 cm in length and the short diameter of lymph node was less than or equal to 10 mm in 11 cases (25 lymph nodes), there were 31, 20, 7 in well-differentiated, moderately differentiated and poorly differentiated, respectively. While in group B, the primary tumor is from 1 cm to 4 cm in length and the short diameter of lymph node was greater than 10 mm in 8 cases (12 lymph nodes), there were 58, 1, 3 in well-differentiated, moderately differentiated and poorly differentiated, respectively (Table 1). As shown in Table 1, there were statistically significant differences in the lymph node metastasis status and differentiation degree between group A and group B (*P* < 0.05), but no statistically significant differences in age, gender, location, pathological type and lesion size (*P* > 0.05).

**Table 1:** General characteristics of patients

Characteristics	Group A (*n* = 58)	Group B (*n* = 62)	*P*
	Number (%)	Number (%)	
**Age**			0.996
< 50	9(15.52)	10 (16.13)	
50 ~ 60	33 (56.90)	35 (56.45)	
> 60	16 (27.58)	17 (27.42)	
**Gender**			0.765
Female	20 (34.48)	23 (37.10)	
Male	38 (65.52)	39 (62.90)	
**Location of the primary lesion**			0.971
Right upper lobe	13 (22.41)	15 (24.19)	
Right middle lobe	2 (3.45)	3 (4.84)	
Right lower lobe	11 (18.97)	13 (20.97)	
Left upper lobe	13 (22.41)	14 (22.58)	
Left lower lobe	19 (32.76)	17 (27.42)	
**Histology**			0.413
Adenocarcinoma	33 (56.90)	33 (53.22)	
Squamous carcinoma	24 (41.38)	26 (41.94)	
Large cell neuroendocrine carcinoma	0 (0.00)	2 (3.23)	
Large cell undifferentiated carcinoma	1 (1.72)	0 (0.00)	
Sarcomatoid carcinoma	0 (0.00)	1 (1.61)	
**Differentiation degree**			0.000
Well-differentiated	31 (53.45)	58 (93.55)	
Moderately differentiated	20 (34.48)	1 (1.61)	
Poorly differentiated	7 (12.07)	3 (4.84)	
**Lymph node metastasis**			0.000
pN0	0 (0.00)	62 (100.00)	
pN1	23 (39.66)	0 (0.00)	
pN2	35 (60.34)	0 (0.00)	
pN3	0 (0.00)	0 (0.00)	
**T staging**			0.658
pT1	23 (39.66)	28 (45.16)	
pT2	22 (37.93)	24 (38.71)	
pT3	13 (22.41)	10 (16.13)	
pT4	0 (0.00)	0 (0.00)	

### The microvessel parameters and CT perfusion parameters between group a and group B

All parameters, including the microvessel parameters (MVD, LVN, LVP and LVA) and CT perfusion parameters (BF, BV, MTT and PMB) were underwent consistency test within inter-observer and intra-observer. The Kappa value of the perfusion parameters within inter-observer and intra-observer is 0.86 and 0.81 respectively, and the Kappa value of the microvessel parameters within inter-observer and intra-observer is 0.85 and 0.82 respectively.

**Fig. 1:**
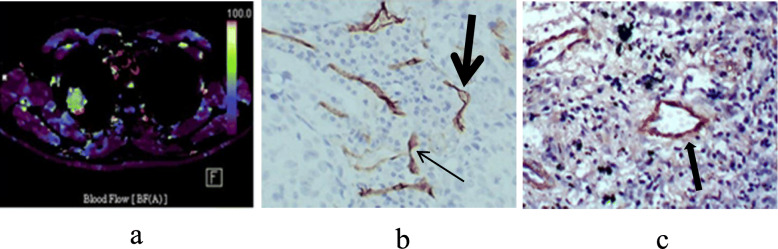
A squamous cell carcinoma at the upper lobe of right lung with right hilar lymph node metastasis. (**a**) Functional map of perfusion showed that blood flow value was low (48.36 ml/100 ml/min). (**b**) CD34 staining showed microvessels with luminal vessels (thick arrow) and without luminal vessels (thin arrow) and the latter accounted for the main part (× 200). (**c**) SMA staining showed fewer microvessels covered with completed layers of smooth muscle cells (arrows) (× 200)

**Fig. 2:**
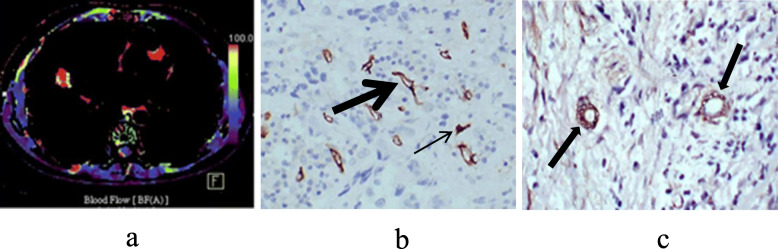
An adenocarcinoma at the middle lobe of right lung without regional lymph node metastasis. (**a**) Functional maps of perfusion show that blood flow value was high (105.23 ml/100 ml/min). (**b**) CD34 staining showed microvessels with luminal vessels (thick arrow) and without luminal vessels (thin arrow) and the former accounted for the main part (× 200). (**c**) SMA staining showed more microvessels covered with completed layers of smooth muscle cells (arrows) (× 200)

The microvessel parameters and CT perfusion parameters of group A and group B were compared. The result showed that group A presented significantly lower LVA, BF and higher MTT, PMB than group B (*P* = 0.027, 0.006, 0.011 and 0.048, respectively). There were no significant difference in BV, LVN, LVP and MVD (*P* > 0.05) (Table 2, Figs. 1 and 2).

**Table 2:** Difference of parameters between group A and group B

Parameters	Group A	Group B	*Z*	*P*
BF (ml/100 ml/min)	66.13 ± 30.15	115.57 ± 80.21	−2.710	0.006
BV (ml/100 ml)	8.58 ± 4.31	9.21 ± 3.54	−0.728	0.467
MTT(s)	15.85 ± 15.92	6.10 ± 2.57	−2.667	0.011
PMB (ml/100 ml/min)	21.18 ± 29.87	20.59 ± 7.91	−1.959	0.048
MVD (strip/field)	67.21 ± 46.69	70.85 ± 50.14	−0.447	0.637
LVN (strip/field)	6.70 ± 3.10	8.31 ± 3.87	−1.268	0.218
LVA (μm^2^/field)	4617.65 ± 1435.67	6541.37 ± 3235.76	−2.310	0.027
LVP (μm/field)	718.71 ± 216.75	942.09 ± 418.39	−1.951	0.052

*Abbreviations*: *BF* blood flow, *BV* blood volume, *LVA* luminal vascular area, *LVN* luminal vascular number, *LVP* luminal vascular perimeter, *MTT* mean transit time, *MVD* microvessel density, *PMB* permeability

### Relationship of CT perfusion parameters with microvessel parameters

Correlation analysis showed that BF was correlated with LVA and LVP (r = 0.335, 0.383, respectively; *P* = 0.031, 0.012, respectively), while BV, MTT and PMB were not correlated with LVN, LVA and LVP (*P* > 0.05). All the perfusion parameters were not correlated with MVD (*P* > 0.05) (Table 3).

**Table 3:** Relationship of CT perfusion parameters with microvessel parameters

Parameters	BF		BV		MTT		PMB	
	*r*	*P*	*r*	*P*	*r*	*P*	*r*	*P*
MVD	0.151	0.371	−0.126	0.431	−0.261	0.110	0.001	0.998
LVN	0.229	0.140	0.071	0.650	−0.051	0.761	0.052	0.750
LVA	0.335	0.031	0.160	0.313	−0.216	0.169	0.163	0.296
LVP	0.383	0.012	0.259	0.091	−0.138	0.379	0.070	0.679

*Abbreviations*: *BF* blood flow, *BV* blood volume, *LVA* luminal vascular area, *LVN* luminal vascular number, *LVP* luminal vascular perimeter, *MTT* mean transit time, *MVD* microvessel density, *PMB* permeability

### The efficiency of CT perfusion parameters in diagnosing regional lymph node metastasis of NSCLC

**Fig. 3:**
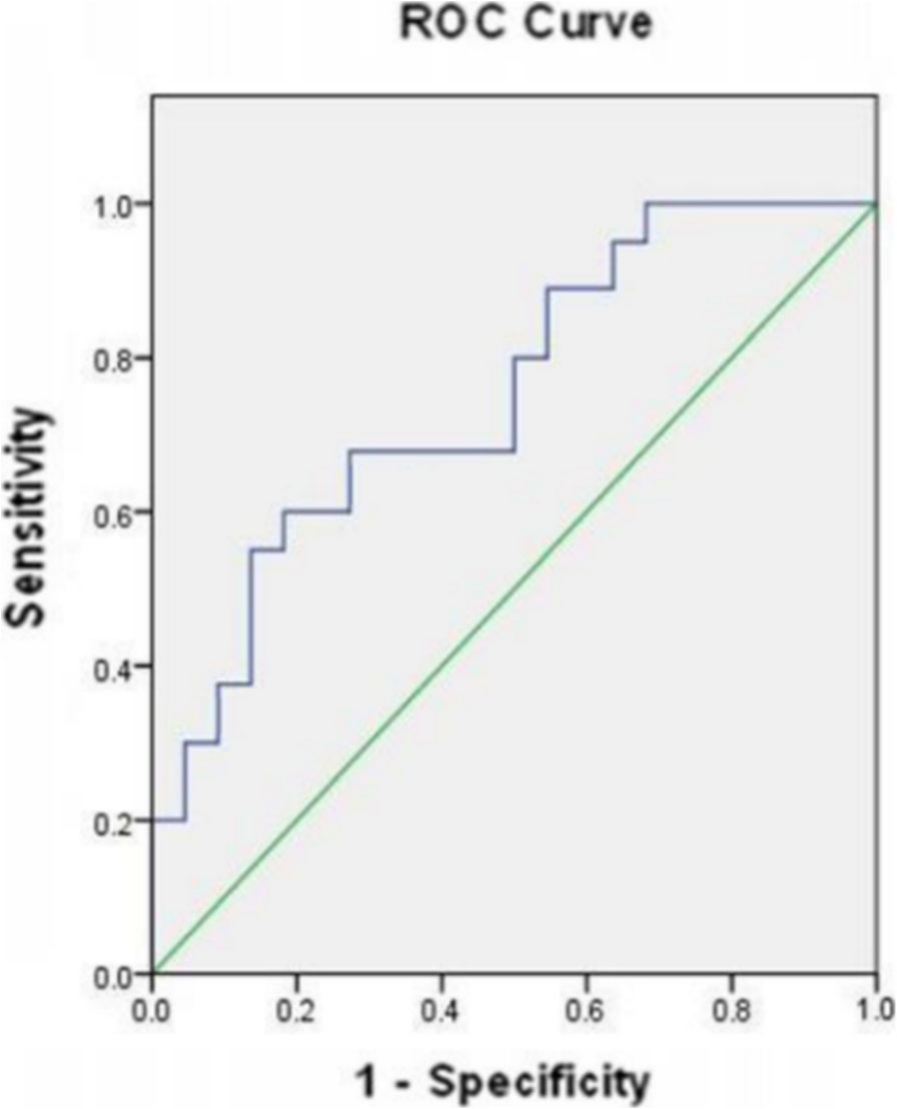
ROC curve of BF in predicting regional lymph node metastasis of NSCLC

According to the above results, the CT perfusion parameter BF, which is different between group A and group B and correlated with the luminal vascular parameters was selected as the index to predict NSCLC with or without regional lymph node metastasis. ROC was used to test the ability of BF to diagnose regional lymph node metastasis of NSCLC. The area under ROC curves (AUC) for BF was 0.746 (*P* < 0.05). According to the ROC curve analysis, when BF < 85.16 ml/100 ml/min as a cutoff point to predict regional lymph node metastasis of NSCLC, the sensitivity, specificity, accuracy, positive predictive value and negative predictive value were 60.8, 81.7, 71.5, 75.6 and 69.5% respectively (Fig. 3).

## Discussion

### Tumor angiogenesis and regional lymph node metastasis of NSCLC

Tumor angiogenesis is an important factor affecting tumor growth, invasion, metastasis and prognosis [12–14]. The lymph node metastasis of tumor cells has three common ways: (i) Tumor cells directly invade the lymphatic vessels; (ii) Tumor cells directly invade into the micro vessels, and then through the incomplete basement membrane into the tumor stroma, and then enter the lymphatic vessels leading to lymph node metastasis; (iii) Tumor cells that entered into the blood circulation may also enter into the stroma of tumor or tissue with blood flow and cause lymph node metastasis. Obviously, in the process, the value of tumor angiogenesis is significant [14, 15]. It was reported that lymph node metastasis of cervical cancer was closely related to tumor angiogenesis [8]. This study also showed that regional lymph node metastasis of NSCLC was related to the luminal vascular parameters, and the luminal vascular parameter LVA in group A are lower than group B, while no correlation with MVD. We believe that regional lymph node metastasis of NSCLC is more closely associated with the luminal vascular, and because of MVD including the vessels with and without lumen [16], the different proportion of them may cause the relationship between MVD and lymph node metastasis different too. Besides, the microvascular wall is mostly thin-walled and fissured, and even if some of the lumen is formed, it is also no function. Therefore, although MVD is considered to be a reliable indicator of tumor angiogenesis [17, 18], luminal vascular parameters may be a better indicator for the evaluation of tumor biological behavior. But the results of this study show that there were no significant difference in LVN and LVP, we found that no matter in group A or group B, the morphology of vascular lumen was inconsistent. In group A, the lumen was mostly oval, while in group B, the blood vessels tended to be more round. Our analysis may be due to two reasons. First, because of the high density of tumor cells in group A, the interstitial space is small. The second reason is that the low maturity of vascular smooth muscle lead to the lumen lack of tension.

### The current situation of preoperative assessment of NSCLC with lymph node metastasis

At present, CT and PET-CT are the main non-invasive methods for preoperative assessment of NSCLC with lymph node metastasis. The size of lymph nodes is the main basis of CT in judging regional lymph node metastasis of NSCLC. The short diameter of lymph node is usually 10 mm as the threshold for the diagnosis of lymph node metastasis, but it is easy to lead to false positive and false negative [3, 4]. Moreover, the metastatic lymph nodes less than 10 mm in short diameter were also easily misdiagnosed in surgical operation and pathological examinations. This study showed that the short diameter of lymph node was less than or equal to 10 mm in 11 cases (25 lymph nodes) of lymph node metastasis group, while greater than 10 mm in 8 cases (12 lymph nodes) of non-lymph node metastasis group. This also shows that it leads easily to false positivity and false negativity, if the size of lymph nodes is used to judge whether the lymph node are metastatic or not. The lymph node greater than 10 mm in short diameter is not necessarily metastasis, but less than 10 mm may also be metastasis.

FDG-PET/CT is currently the most important noninvasive method for diagnosing lymph node metastasis. Maximal standardized uptake value (SUVmax) is a good index marker for the diagnosis of metastatic lymph nodes, but there is no consensus on the optimal threshold of SUVmax, and some non-metastatic lymph nodes can also have high uptake of 18 F-FDG. it may also lead to false positive and false negative [19, 20]. In addition, due to the fact that PET/CT equipment and inspection fees are very expensive, it has not been widely used, especially in China.

### The value of CT perfusion parameters in diagnosing regional lymph node metastasis of NSCLC in pre-operation

CTPI can provide qualitative and quantitative hemodynamic information, and it can reflect noninvasively the angiogenesis of tumor [9, 21, 22]. It has important application value in research of tumor. This study showed that CT perfusion parameter BF was correlated with the luminal vascular parameters LVA. All the perfusion parameters were not correlated with MVD. BF refers to the flow rate of blood in unit time and volume. That is to say, the larger the LVA, the larger the blood flow rate and the blood volume of per unit volume and time. This demonstrates that the dual source CT perfusion parameters can indicate the luminal vessels of tumor, but as for MVD is uncertain. This may also be associated with the microvessels including some non-functional vessels.

It was reported that CTPI plays an important role in evaluating lymph node metastasis of cancer in pre-operation [23, 24]. This study showed that the dual source CT perfusion parameters are related to the luminal vascular parameters of NSCLC, while the luminal vascular parameters are related to regional lymph node metastasis of NSCLC, which provides a theoretical basis for the evaluation of regional lymph node metastasis of NSCLC by CT perfusion parameters. In this study, CT perfusion parameter BF of NSCLC group with regional lymph node metastasis was lower than that of NSCLC group without regional lymph node metastasis. This showed that the dual source CT perfusion parameter BF have certain value in predicting regional lymph node metastasis of NSCLC. Our analysis may be due to following reasons. First, we found that there were more poorly differentiated tumors in group A than in group B. In group A, the lumen was mostly oval, while in group B, the blood vessels tended to be more round, so BF of group A was lower. The second reason is that the more poorly differentiated tumors, the more obvious the destruction of blood vessels was, which was more favorable for the cancer cells to enter the interstitial tissue to have lymph node metastasis, but because of the serious destruction of blood vessels, caused the low blood flow. In addition, the immature blood vessels in poorly differentiated tumors are relatively more and disordered, which also affects the blood flow rate of the tissues to a certain extent, and the proliferation speed of poorly differentiated tumor cells is fast, and the situation of tissue ischemia and hypoxia is more. In this study, BV, MTT and PS there are no correlation with microvascular parameters. BV there is no difference in between group A and group B, therefore, we speculated that blood volume can only reflect the total amount of blood contained in the lesion, but its value in reflecting its biological behavior is limited. PS is not only related to the integrity of endothelial cells, but also to the pressure balance between plasma and tissue fluid. Poorly differentiated tumors have relatively severe damage to tumor blood vessels, which may increase the permeability of blood vessels. However, the rapid proliferation of poorly differentiated tumor cells will leads to the increase of tissue fluid pressure. Therefore, we believe that PS are uncertain in the evaluation of lymph node metastasis. MTT there is difference in between group A and group B, but there are no correlation with microvascular parameters, therefore, we have no reliable theoretical basis for predicting regional lymph node metastasis of NSCLC by MTT. MTT may be a potential indicator, but we need to deepen our understanding of tumor blood vessels to confirm this conclusion.

ROC curve was used to evaluate the value of the dual source CT perfusion parameter BF in predicting regional lymph node metastasis of NSCLC. The result showed that BF was valuable in predicting regional lymph node metastasis of NSCLC in pre-operation. And the possibility of NSCLC with regional lymph node metastasis may be suggested when BF < 85.16 ml/100 ml/min. For these patients, the regional lymph node dissection should be performed more carefully, systematically and extensively to avoid missing the small metastatic lymph nodes.

## Conclusion

This study showed that the luminal vascular parameters were the important indexes for the evaluation of tumor angiogenesis and were related to the regional lymph node metastasis of NSCLC. Flash dual source CT perfusion imaging can non-invasively indicate the luminal vascular structure of tumor and BF can be used as one of the important indexes in predicting regional lymph node metastasis of NSCLC in pre-operation. So the dual source CT perfusion imaging can be an effective supplement of traditional morphological image in diagnosing regional lymph node metastasis of NSCLC.

Small sample size, a scanner from a single vendor and single-center study are the main limitations of this study. Therefore, further work is to expand the sample size and conduct multi-center research to improve the value of the CT perfusion parameters in predicting regional lymph node metastasis of NSCLC.

## Data Availability

All data generated or analyzed during this study are included in this article and its supplementary information files.

## Abbreviations

AUC: Area under the curve
BF: Blood flow
BV: Blood volume
CT: Computed tomography
CTDIvol: Volume computed tomography dose index
CTPI: Computed tomography perfusion imaging
DLP: Dose length product
LVA: Luminal vascular area
LVN: Luminal vascular number
LVP: Luminal vascular perimeter
MTT: Mean transit time
MVD: Microvessel density
NSCLC: Non-small cell lung cancer
PMB: Permeability
ROC: Receiver operating characteristic
ROI: Region of interest
SMA: Smooth muscle action
SUVmax: Maximal standardized uptake value
VPCT: Volume perfusion computed tomography
